# Study on kinetics and thermsodynamics of municipal solid waste incineration fly ash in air and N_2_ atmospheres

**DOI:** 10.1371/journal.pone.0323729

**Published:** 2025-05-14

**Authors:** Yegui Wang, Weifang Chen, Na Zhao, Yifan Chen, Baoqing Deng

**Affiliations:** School of Environment and Architecture, University of Shanghai for Science and Technology, Shanghai, China; Universiti Teknologi Petronas: Universiti Teknologi PETRONAS, MALAYSIA

## Abstract

This study attempted to investigate the thermal behavior and reaction mechanisms of municipal solid waste incineration fly ash under air and N_2_. Mass loss patterns at temperatures from 30ºC to 1100ºC were obtained through thermogravimetric analysis. Based on mass loss patterns, the behavior of fly ash under high temperature was divided into three stages. Mass loss in Stage I (30ºC-500ºC) amounted to 3.0%-6.2%. The majority of mass loss concentrated in Stage II (500ºC-800ºC) and Stage III (800ºC-1100ºC). Kinetic parameters of fly ash in Stage II and Stage III were evaluated using Flynn-Wall-Ozawa (FWO), Kissinger-Akahira-Sunose (KAS), and Friedman methods. By comparison, the iso-conversional FWO method exhibited the highest correlation coefficient with R^2^ > 0.99. Activation energy (E) values in Stage II calculated via the FWO method indicate that reaction in air showed considerably higher hurdle (E = 171.11 kJ/mol) than reaction in N_2_ (E = 124.52 kJ/mol). This difference was partly attributed to the presence of carbonation process in air. In contrast, E values in Stage III were similar with E of 373.38 kJ/mol in air and 382.25 kJ/mol in N_2_. Mechanistic analysis via the Coats-Redfern (CR) model, employing 15 kinetic functions, identified dominant mechanisms of one-dimensional diffusion and contracting sphere for Stage II in air and N_2_ respectively. At the same time, three-dimensional diffusion could best explain the reaction mechanism in Stage III in both air and N_2_. Moreover, calculations of thermodynamic parameters (ΔH, ΔG, and ΔS) revealed that major reactions of fly ash during thermal treatment were endothermic and non-spontaneous, with Stage III exhibiting heightened complexity. This multi-stage characterization elucidates the degradation mechanisms of fly ash under varying thermal conditions and provides useful insight into the fly ash thermal treatment processes.

## 1. Introduction

The global production of municipal solid waste has been growing steadily and is expected to reach 3.4 billion tons by 2050 [1]. Landfill and incineration are two of the most common methods of disposal. However, landfill is land-intensive while gaseous products and leachate from landfill facilities present great environmental challenges [2]. Incineration is renowned for its ability to effectively reduce mass (about 70%) and volume (about 90%) of municipal solid waste. But incineration also has its problems. One of them is the generation of fly ash. Fly ash is classified as hazardous waste in many countries due to its contents of toxic organics, heavy metals and salts [3]. On the other hand, fly ash could also be a valuable resource as it is rich in chemicals such as CaCO_3_, SiO_2_, and Al_2_O_3_. Therefore, the main focus on fly ash is effective treatment, safe disposal or cost-effective resource recycling.

Thermal treatment, stabilization/solidification, and separation are the main treatment methods for municipal solid waste incineration fly ash. Compared with the other two methods, thermal treatment has certain advantages in reducing volume, stabilizing heavy metals and decomposing toxic organics [4]. Studies have shown that thermal treatment around 400ºC can remove 95% of dioxins from fly ash [5], while treatment at 800ºC can effectively reduce heavy metal leaching [6]. Recent advancements in thermal treatment technologies have focused on the detoxification effects and resource utilization pathways. For instance, co-treating fly ash with iron ore has been shown to stabilize heavy metals [7], while high-temperature treatments enable the extraction of SiO_2_ [8]. Additionally, fly ash has been successfully utilized in the preparation of cementitious materials [9] and ceramic products [10]. Due to its high efficiency and versatility, thermal treatment has gained considerable attention. Many studies have focused on the behavior of fly ash during thermal treatment.

Gu et al. [11] compared the effects of atmospheric conditions on thermal treatment. Their results showed that mass loss of fly ash followed the order of CO_2_ > N_2_ > air. Lane et al. [12] further investigated the relationship between the volatility of elements and atmosphere of thermal treatment. Elements such as K, Na, Pb, Cd, and Cu exhibited higher volatility in oxidative atmospheres. By comparison, Zn, Sb, Sn, and Bi were more readily released in reducing atmospheres. Importantly, the migration and transformation of heavy metals during thermal treatment are also strongly influenced by the choice of atmosphere [13]. These findings underscore the critical role of atmospheric conditions in determining the outcomes of thermal treatment, as they directly affect both the volatilization behavior of elements and the mass loss of fly ash.

Thermal treatment is a prevalent technology for fly ash management. Kinetic and thermodynamic analyses of these processes remain understudied. Kinetic and thermodynamic analyses enable the prediction of reaction feasibility and limitation, and mechanistic pathways [14,15], while providing essential guidance for reactor design optimization and industrial-scale parameterization [16]. Therefore, systematic investigations into fly ash’s thermal behavior under various conditions is necessary.

Thermogravimetric analysis (TG) stands as a pivotal tool in the investigation of the thermal behavior of solid materials [17,18]. By varying temperature, atmosphere, heating rate and reaction time, alterations in solid mass or conversion ratio could be obtained. Kinetic and thermodynamic parameters of solid materials during thermal treatment can then be computed by model-free and model-fitting methods. For instance, Yousef et al. [19] and Loy et al. [20] similarly applied model-free methods including Friedman, FWO and KAS to estimate the activation energy of mango seed shells and rice hull under high temperature. In addition, model-fitting methods such as the CR method can be employed to determine the reaction mechanism and pre-exponential factors based also on TG data [21,22]. That is, TG analysis results were versatile. They could be used to capture the thermodynamic and kinetic characteristics of the thermal behavior of solids. By fitting the data into various reaction mechanism models, they could also be used to elucidate the reaction mechanisms. For example, Chen et al. [23] utilized the determined kinetic parameters to compute enthalpy changes (ΔH), Gibbs free energy (ΔG), and entropy (ΔS) to assess the feasibility, direction, and energy requirements for thermal treatment of waste medical surgical mask rope. Despite these established applications in fuel and energy research, fly ash thermal treatment mechanisms remain insufficiently characterized. This deficiency not only limited a thorough understanding of fly ash thermal treatment mechanisms but also impeded the development and practical application of related technologies.

This study attempted to investigate the thermal behavior of fly ash in air and N_2_ via thermogravimetric analysis. Three model-free methods, including the Friedman method, FWO method, and KAS method, were used to determine the reaction activation energy at different conversion ratios. One model-fitting method (CR method) was employed to determine the reaction-dominating model and pre-exponential factor. The accuracy of the obtained model was evaluated by comparing the calculated and experimental results. In addition, thermodynamic parameters like Gibbs free energy, enthalpy, and entropy were also calculated and discussed. This research endeavored not only to enhance the understanding of the thermal treatment of fly ash but also to contribute insights for process optimization.

## 2. Materials and methods

### 2.1 Raw materials

The fly ash used in this study was collected from a municipal solid waste incineration plant located in Zhejiang Province, China. To ensure sample homogeneity, composite sampling was carried out continuously for 14 days, and the collected samples were thoroughly mixed. The fly ash was then ground and sieved through a 100-mesh sieve then dried at 105ºC for 24 h before use.

### 2.2 Fly ash characterization

A TG/DSC simultaneous thermal analyzer (TG/DSC, PE STA8000, USA) was used to explore the thermal behavior of fly ash under air and N_2_ atmosphere. About 10 mg of fly ash was calcinated from 30ºC to 1100ºC in an alumina crucible. The heating rates were set at 10ºC/min, 20ºC/min, and 30ºC/min, respectively. Gas flow rates were constant at 80 mL/min.

Proximate analysis of fly ash was obtained according to ASTM standard method E1131-08. X-ray Diffraction (XRD, Rigaku UItima IV, Japan) was employed to characterize the crystalline phases, with Cu-Kα radiation at 40 KV and 30 mA as 2θ was ranged from 5° to 90°. Chemical compositions and micromorphology of fly ash were determined via X-ray Fluorescence Spectroscopy (XRF-1800, Shimadzu, Japan) and Scanning Electron Microscope (SEM, Zeiss Gemini 300, Germany).

### 2.3 Kinetic and thermodynamics analyses

#### 2.3.1 Estimation of activation energy.

TG/DSC results were used to determine the kinetic models and thermodynamic parameters of fly ash. Heterogeneous system kinetics (Eq. (1)) for solid decomposition reactions were selected.


$$\frac{d\alpha }{dt}=k(T)f(\alpha )$$ (1)

where α is the conversion ratio and is calculated by Eq. (2); t is heating time; T is the reaction temperature and f(α) is the conversion function. k(T) stands for the reaction rate constant and can be represented by the Arrhenius equation as Eq. (3).


$$\alpha =\frac{m_{0}-m_{t}}{m_{0}-m_{\infty }}$$ (2)

$$k(T)=Aexp(-\frac{E}{RT})$$ (3)

where m_0_, m_t_, and m_∞_ are the initial mass, mass at time t, and final mass, respectively; A is the pre-exponential factor; E is the activation energy and R is the universal gas constant (8.314 J/mol/K).

At a constant heating rate (β=dT/dt), Eq. (1) can be rewritten as follows:


$$\frac{d\alpha }{dT}=\frac{A}{\beta }exp(-\frac{E}{RT})f(\alpha )$$ (4)

Friedman method, FWO method, and KAS method were employed to evaluate activation energy at different α [24].

Among them, the Friedman method is an iso-conversional method in differential form. In this method, Eq. (4) is converted to Eq. (5) by taking the logarithm of both sides.


$$\ln\left(\beta \frac{d\alpha }{dT}\right)=\ln\left(\frac{d\alpha }{dt}\right)=\ln\left[Af(\alpha )\right]-\frac{E}{RT}$$ (5)

E is calculated from the slope of a linear relationship between ln (dα/dt) and 1/T.

FWO and KAS are iso-conversional methods in integral form. Eq. (4) is rearranged to obtain Eq. (6) first. The integral form of f(α) is designated as g(α) to obtain Eq. (7). The equation is further transferred to Eq. (8) and Eq. (9) via mathematically simplified FWO and KAS methods respectively [25].


$$\frac{d\alpha }{f(\alpha )}=\frac{A}{\beta }exp(\frac{-E}{RT})dT$$ (6)

$$g(\alpha )=\int_{0}^{\alpha }\frac{d\alpha }{f(\alpha )}=\frac{A}{\beta }\int_{T_{0}}^{T}exp(\frac{-E}{RT})dT$$ (7)

$$\ln\beta =\ln\left(\frac{AE}{Rg(\alpha )}\right)-5.331-1.052(\frac{E}{RT})$$ (8)

$$\ln\left(\frac{\beta }{T^{2}}\right)=\ln\left(\frac{AE}{Rg(\alpha )}\right)-\frac{E}{RT}$$ (9)

Here, E can be calculated from the slopes of Eq. (8) and Eq. (9) by calculating the linearity ln(β) vs. 1/T and ln(β/T^2^) vs. 1/T, respectively.

#### 2.3.2 Determination of reaction mechanism function.

In Eq (7), g(α) is the integral form of f(α) and is related to the reaction mechanism [26]. Depending on the reaction mechanism, g(α) could be expressed in different functions. Table 1 lists fifteen of the most common mechanism functions of solid-state thermal reactions. These functions are divided into 5 categories: chemical reaction, phase boundary reactions, diffusion, random nucleation and nuclei growth, and exponential nucleation.

**Table 1:** Common kinetic mechanism functions of solid-state thermal reactions [27].

Reaction mechanism		f(α)	g(α)
**Chemical reaction**			
**F2**	Second-order	(1-α)^2^	(1-α)^-1^–1
**F3**	Third-order	1/2(1-α)^3^	(1-α)^-2^–1
**Phases boundary reaction**			
**R1**	Contracting disk	1	α
**R2**	Contracting cylinder	2(1-α)^1/2^	1-(1-α)^1/2^
**R3**	Contracting sphere	3(1-α)^2/3^	1-(1-α)^1/3^
**Diffusion**			
**D1**	One-dimensional diffusion	1/(2α)	α^2^
**D2**	Two-dimensional diffusion	[-ln(1-α)]^-1^	α+(1-α)ln(1-α)
**D3**	Three-dimensional diffusion	3/2(1-α)^2/3^[1-(1-α)^1/3^]^-1^	[1-(1-α)^1/3^]^2^
**D4**	Ginstling-Brounshtein equation	3/2[(1-α)^-1/3^–1]^-1^	(1–2α/3)-(1-α)^2/3^
**Random nucleation and nuclei growth**			
**A2**	Avarami-Erofeev (n = 2)	2(1-α)[-ln(1-α)]^1/2^	[-ln(1-α)]^1/2^
**A3**	Avarami-Erofeev (n = 3)	3(1-α)[-ln(1-α)]^2/3^	[-ln(1-α)]^1/3^
**A4**	Avarami-Erofeev (n = 4)	4(1-α)[-ln(1-α)]^3/4^	[-ln(1-α)]^1/4^
**Exponential nucleation**			
**P2**	Power law (n = 1/2)	2α^1/2^	α^1/2^
**P3**	Power law (n = 1/3)	3α^2/3^	α^1/3^
**P4**	Power law (n = 1/4)	4α^3/4^	α^1/4^

In this study, the CR approximation method, a model-fitting method based on g(α), was used to determine the reaction model as well as the pre-exponential factor [28]. Via CR approximation, Eq. (7) is simplified to Eq. (10).


$$\ln\frac{g(\alpha )}{T^{2}}=\ln\frac{AR}{\beta E}-\frac{E}{RT}$$ (10)

By comparing the goodness-of-fit between ln(g(α)/T^2^) and 1/T of the TG data with different g(α) functions, the best-fit model of the reaction mechanism was selected. E and A values were estimated from slope and intercept, respectively.

#### 2.3.3 Estimation of thermodynamic parameters.

The thermodynamic parameters, ΔG, ΔH, and ΔS, were calculated from Eq. (11) to Eq. (13) based also on E values obtained and TG analysis results [29].


$$\Delta G=E+RT_{p}\ln\frac{K_{B}T_{p}}{hA}$$ (11)

$$\Delta H=E-RT_{\alpha }$$ (12)

$$\Delta S=(\Delta H-\Delta G)/T_{p}$$ (13)

where T_p_, K_B_ and h are the peak temperature of DTG curve, Boltzmann constant (1.381 × 10^-23^ J/K) and Plank constant (6.626 × 10^-34^ J ∙ s), respectively. T_α_ is the temperature at the α.

## 3. Results and discussion

### 3.1 Characteristics of fly ash

The results of proximate analysis and chemical composition of the fly ash are detailed in Table 2. Fly ash contained 3.1 wt% of volatile carbon and 9.9 wt% of fixed carbon, with the majority being ash. This was attributed to the high-temperature combustion in waste incineration plants where volatile carbon was effectively consumed. XRF results revealed that the relatively predominant elements were Ca, Cl, Na, K, and S. The high content of Ca was related to the CaO or Ca(OH)_2_ sprayed to neutralize acidic gas for flue gas treatment [30]. The 12.2 wt% of Cl most likely stemmed from chlorine-containing plastic products or kitchen wastes [31]. In addition, there were 3.8 wt% of SiO_2_, 3.3 wt% of Fe_2_O_3_, and 1.5 wt% of Al_2_O_3_.

**Table 2:** Proximate analysis and XRF results of fly ash.

Proximate analysis (wt%)			XRF analysis (wt%)							
Volatile	Fixed carbon	Ash	CaO	Cl	K_2_O	Na_2_O	SO_3_	SiO_2_	Fe_2_O_3_	Al_2_O_3_
3.1	9.9	87.0	47.4	12.2	8.1	7.8	6.7	3.8	3.3	1.5

**Fig 1:**
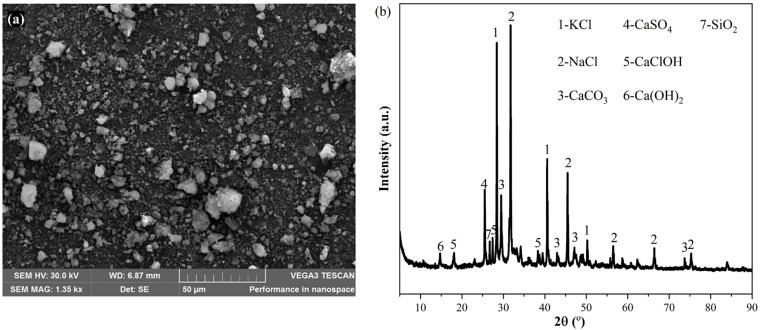
(a) SEM image; (b) XRD pattern of fly ash.

SEM and XRD analysis were next performed to further illustrate the micromorphology and crystalline phases of fly ash. Fig 1 (a) is the SEM image of the fly ash. Fly ash is composed of particles of different sizes and irregular shapes. XRD analysis (Fig 1 (b)) revealed the presence of calcite (CaCO_3_), anhydrite (CaSO_4_), calcium chloride hydroxide (CaClOH), portlandite (Ca(OH)_2_), sylvite (KCl), halite (NaCl) and silica (SiO_2_). Both XRF and XRD analysis showed that fly ash had an abundance of chemicals including Ca-compounds, salts of chloride, carbonate, sulfate and inorganic oxides. The complexity in composition may imply complex chemical reactions during thermal treatment.

### 3.2 Thermogravimetric analysis

**Fig 2:**
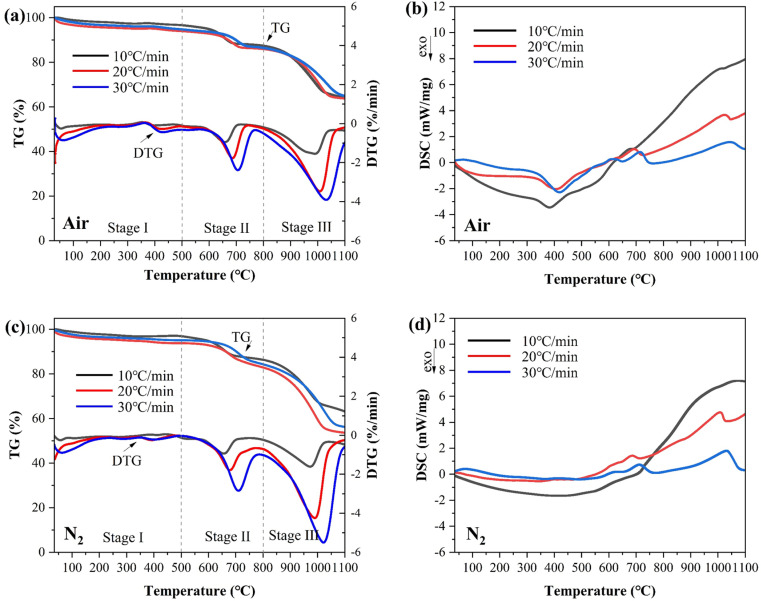
(a) TG and DTG in air; (b) DSC in air; (c) TG and DTG in N_2_ and (d) DSC in N_2_ of fly ash at heating rates of 10ºC/min, 20ºC/min, and 30ºC/min.

Thermogravimetric analysis was used to understand the thermal behavior of fly ash. Fig 2 showcases the TG, DTG, and DSC curves of fly ash with heating rates of 10ºC/min, 20ºC/min, and 30ºC/min under air and N_2_ atmosphere, respectively. As shown in Fig 2(a) and (c), the total mass losses were 35.22%-36.28% and 36.83%-46.38% in air and N_2,_ respectively. Mass losses before 500ºC both in air and N_2_ amount to only 3.36%-6.05% and 3.05%-6.22% indicating that changes in fly ash did not occur until relatively high temperature. The 1.92%-4.32% and 2.20%-4.38% mass loss in air and N_2_ occurred at around 30ºC and 200ºC could be attributed to the loss of moisture [32]. In addition, a minor peak at 400ºC was observed from TG in air. This could be explained by the combustion of carbon to CO_2_ [11]. The peak was absent from TG in N_2_. The majority of mass loss was between 500ºC-1100ºC as manifested in two major peaks around 656ºC-710ºC and 973ºC-1031ºC in DTG curves.

About 8%-11% of fly ash was lost between 500ºC and 800ºC. This loss was caused by the decomposition of compounds such as CaClOH and CaCO_3_ [33]. Peaks in DSC curves in Fig 2(b) and (d) around 700ºC indicate that these reactions were endothermic. Mass losses of 21.54%-29.28% were observed at 800ºC-1100ºC. At this temperature range, it is believed that most of the original compounds were either decomposed, volatilized or melted. Zhao et al. [34] reported that reactions in this temperature range included volatilization of chloride salts, disintegration of sulfates, and production of silicates. Overall, TG and DTG patterns of fly ash in air and N_2_ showed similar patterns. In light of the mass loss behavior, thermal processes can be divided into three stages, namely, Stage I (30ºC-500ºC), Stage II (500ºC-800ºC) and Stage III (800ºC-1100ºC).

TG results in Fig 2 further revealed that the heating rate also affected the thermal behavior. TG, DTG, and DSC curves tended to migrate to higher temperatures as the heating rate increased. This trend was ascribed to the fact that the system experienced reduced dwell time at higher heating rates, leading to insufficient thermal energy accumulation [24]. Consequently, a higher reaction temperature was required to compensate for the accelerated thermal input and to ensure enough heat energy for the chemical reaction. While variations in heating rates caused the peak temperature and reaction rate to change, they did not alter the thermal profile of the entire system [35].

### 3.3 Estimation of activation energy by model-free methods

Activation energy E represents an energy barrier, i.e., the minimum energy required for a reaction to occur. The lower the E value, the easier it is for the reaction to occur [23]. E values of fly ash at different stages of TG thermal behavior were calculated via FWO, KAS, and Friedman methods in this study. The α range was selected from 0.1 to 0.9. S1 and S2 Fig. in Supplementary Materials display the plots of lnβ, ln (β/T^2^), and ln (dα/dt) versus 1/T in air and N_2_, respectively. Correlation coefficient R^2^ ranged from 0.626–1.000 indicating good linearity at various α values. The values of E were calculated according to Eq. (5)-Eq. (9). Tables 3 and 4 are the E and R^2^ values at Stage II and Stage III of TG analysis in air and N_2,_ respectively.

**Table 3:** The values of E and R^2^ of fly ash thermal treated in air based on FWO, KAS, and Friedman methods.

Stage	α	T* (ºC)	FWO		KAS		Friedman	
			E (kJ/mol)	R^2^	E (kJ/mol)	R^2^	E (kJ/mol)	R^2^
**II**	0.1	591	188.57	0.992	184.12	0.991	167.00	0.989
	0.2	610	171.55	0.999	165.85	0.998	151.45	0.977
	0.3	636	183.30	0.999	177.24	0.999	134.60	0.992
	0.4	655	166.30	0.998	159.58	0.998	135.66	0.986
	0.5	667	162.42	0.996	155.28	0.995	158.66	0.984
	0.6	677	163.50	0.993	156.24	0.992	166.68	0.979
	0.7	686	167.67	0.991	160.46	0.989	192.59	0.956
	0.8	695	170.05	0.986	162.80	0.983	168.97	0.936
	0.9	711	166.60	0.992	158.94	0.990	190.84	0.986
	Average		**171.11**	**0.994**	**164.50**	**0.993**	**173.97**	**0.976**
**III**	0.1	862	356.13	1.000	355.82	1.000	443.83	0.989
	0.2	907	391.57	0.997	392.40	0.997	392.62	1.000
	0.3	931	391.68	0.994	392.05	0.994	348.73	0.979
	0.4	957	366.09	0.999	364.77	0.999	329.08	0.979
	0.5	972	371.41	0.997	370.06	0.997	332.76	0.941
	0.6	995	364.62	0.972	362.66	0.968	372.09	0.988
	0.7	1011	355.51	0.983	352.80	0.981	350.73	0.982
	0.8	1024	368.45	0.999	366.15	0.998	393.89	0.985
	0.9	1035	394.92	0.980	393.69	0.978	493.35	0.988
	Average		**373.38**	**0.991**	**372.27**	**0.990**	**384.12**	**0.981**

*T (ºC): the reaction temperature at heating rate of 20ºC/min.

**Table 4:** The values of E and R^2^ of fly ash thermal treated in N_2_ based on FWO, KAS, and Friedman methods.

Stage	α	T* (ºC)	FWO		KAS		Friedman	
			E (kJ/mol)	R^2^	E (kJ/mol)	R^2^	E (kJ/mol)	R^2^
**II**	0.1	582	86.89	0.974	77.51	0.964	71.40	0.990
	0.2	625	105.87	0.999	96.63	0.998	140.65	0.989
	0.3	648	120.61	0.999	111.67	0.999	133.32	0.998
	0.4	664	127.60	0.999	118.76	0.999	147.02	0.997
	0.5	677	133.77	0.999	125.03	0.999	148.24	1.000
	0.6	689	138.03	0.999	129.32	0.999	149.78	0.983
	0.7	703	139.46	0.997	130.66	0.996	130.31	0.760
	0.8	725	134.78	0.951	125.52	0.938	130.82	0.626
	0.9	752	133.72	0.996	123.84	0.995	221.80	0.987
	Average		**124.52**	**0.990**	**115.44**	**0.988**	**141.48**	**0.925**
**III**	0.1	857	376.24	0.986	377.11	0.985	444.32	0.996
	0.2	896	377.62	0.999	377.85	0.999	338.92	0.954
	0.3	925	391.45	0.998	391.97	0.998	350.98	0.957
	0.4	944	385.55	0.993	385.38	0.992	358.97	0.907
	0.5	965	407.24	0.997	407.92	0.997	388.41	0.973
	0.6	980	391.79	0.998	391.38	0.997	347.92	0.958
	0.7	993	386.00	0.988	385.02	0.987	366.95	0.952
	0.8	1007	386.13	0.979	384.90	0.977	458.91	0.973
	0.9	1036	338.23	0.992	334.09	0.991	443.60	0.971
	Average		**382.25**	**0.992**	**381.74**	**0.992**	**388.77**	**0.959**

*T (ºC): the reaction temperature at heating rate of 20ºC/min.

As shown in Table 3 and Table 4, the thermal behavior of fly ash could be explained as single-step kinetic reactions because the variation of E at 0.1 ≤ α ≤ 0.9 was not significant [36]. In addition, the FWO model consistently showed higher correlations (R^2^ values) in linearity than the KAS and Friedman methods at all α values, indicating that the FWO method explained the experimental data better. Based on the FWO method, the average E values in Stage II and Stage III of fly ash in air were 171.11 kJ/mol and 373.38 kJ/mol while those in N_2_ were 124.52 kJ/mol and 382.25 kJ/mol, respectively.

Notably, in Stage II (500ºC-800ºC), the activation energy of fly ash in N_2_ was lower than that in air. This could mean that reactions in N_2_ were easier to happen and maybe simpler. For example, studies have shown that, in air, the carbon in fly ash was combusted to form CO_2_ [24]. This is also true with the TG results in this study as shown in Fig 2 (a). Thus, CaClOH was carbonated to CaCO_3_ first. CaCO_3_ was next decomposed to CaO and CO_2_ at temperatures between 500ºC and 800ºC [37]. In N_2_, CaClOH underwent a simpler breakdown to CaO or CaCl at temperatures between 550ºC and 800ºC [38]. This is in accordance with the reaction temperature ranges in air (591ºC-711ºC) and N_2_ (582ºC-752ºC). Because of the lack of carbonation, compounds such as CaClOH remained stable at lower temperatures and only decomposed at relatively higher temperatures.

Activation energies in Stage III were quite close between air and N_2_. The same was true of the reaction temperatures range (862ºC-1035ºC in air, 857ºC-1036ºC in N_2_). The similar E values and reaction temperatures indicated that fly ash may undergo similar thermal reactions at high temperatures. It was believed that this stage was characterized by the presence of complex reactions. These prominently involved the volatilization of chlorides and formation of complex compounds (such as mayenite ((CaO)_12_(Al_2_O_3_)_7_), chlorellestadite (Ca_10_(SiO_4_)_3_(SO_4_)_3_Cl_2_) etc.), as expressed in reactions (14)-(16) [39].


$$\text{NaCl/KCl}\to {\text{ Na}}^{\text{+}}\text{/}{\text{K}}^{\text{+}}\text{+}{\text{Cl}}^{\text{-}}$$ (14)

$$\text{12CaO+7}{\text{Al}}_{\text{2}}{\text{O}}_{\text{3}}\to {\text{ (CaO)}}_{\text{12}}{\text{(}{\text{Al}}_{\text{2}}{\text{O}}_{\text{3}}\text{)}}_{\text{7}}$$ (15)

$$\text{6CaO+}{\text{CaCl}}_{\text{2}}\text{+}{\text{3CaSO}}_{\text{4}}\text{+}{\text{3SiO}}_{\text{2}}\to {\text{ Ca}}_{\text{10}}{\text{(}{\text{SiO}}_{\text{4}}\text{)}}_{\text{3}}{\text{(}{\text{SO}}_{\text{4}}\text{)}}_{\text{3}}{\text{Cl}}_{\text{2}}$$ (16)

**Fig 3:**
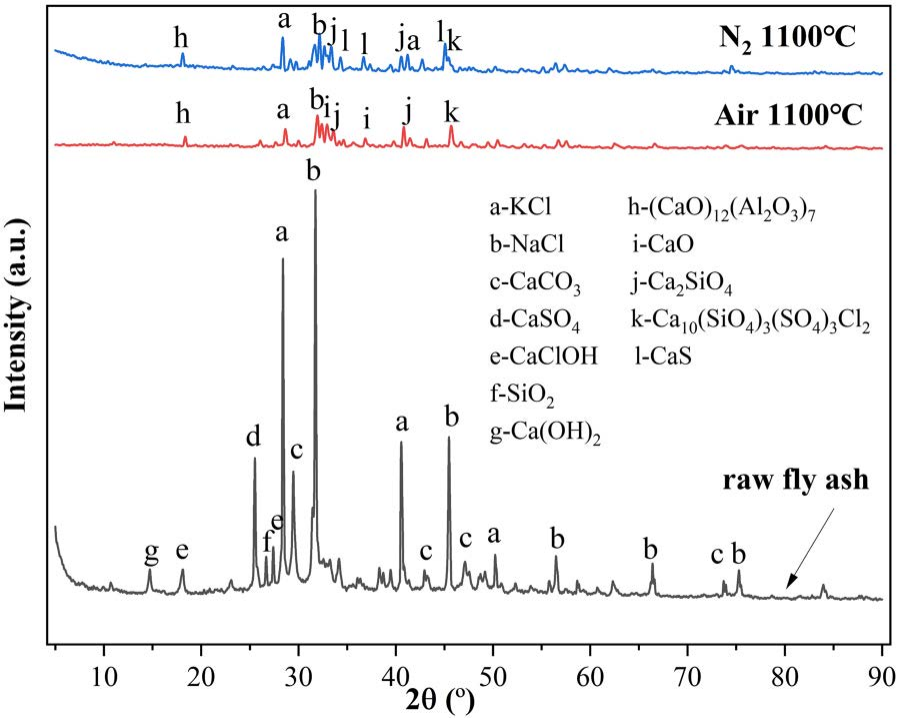
XRD patterns of fly ash before and after TG tests.

As shown in Fig 3, crystal structures of fly ash thermally treated at 1100ºC via XRD revealed significant alterations compared to the original fly ash. The peak intensity of NaCl and KCl dropped sharply and complex crystals like Ca_2_SiO_4_, (CaO)_12_(Al_2_O_3_)_7_, and Ca_10_(SiO_4_)_3_(SO_4_)_3_Cl_2_ emerged at 1100ºC. The XRD results also confirmed the occurrence of intricate reactions in fly ash in this study at elevated temperatures. Therefore, Stage III exhibited much higher E values compared to Stage II to achieve these complex reactions.

### 3.4 The determination of reaction models by model-fitting method

Analysis of mass loss and calculations of activation energies above showed that thermal treatment of fly ash is a complex process involving various mechanisms such as evaporation, decomposition and nucleation [40]. Next, the CR method was used to determine the reaction mechanisms at each stage from TG data. In Eq. (10), g(α) in different forms were fitted with TG data. S1-S4 Tables list the goodness-of-fit (R^2^) and E values calculated from the slopes. TG and DTG results showed that mass loss patterns at different heating rates were quite similar. This is in accordance with the calculated E and R^2^ values which varied little at different heating rates. The average values were used to judge the rationality of the model. A model with the highest R^2^ was chosen as the reaction mechanism for fly ash in this study. Table 5 shows the mean values of kinetic parameters under the optimal reaction model.

**Table 5:** The averages E, R^2^, and A were calculated under the best model.

Stage	Reaction model	g(α)	E (kJ/mol)	A (s^-1^)	R^2^
**Air**					
**II**	One-dimensional diffusion	α^2^	182.31	5.37E + 10	0.98
**III**	Three-dimensional diffusion	[1-(1-α)^1/3^]^2^	386.92	1.57E + 17	0.99
**N**_**2**_					
**II**	Contracting sphere	1-(1-α)^1/3^	127.12	2.21E + 07	0.99
**III**	Three-dimensional diffusion	[1-(1-α)^1/3^]^2^	372.08	2.00E + 16	0.99

For Stage II in air, the highest average R^2^ value of 0.98 was observed in the one-dimensional diffusion model. The integral form g(α)=α^2^ and conversion function f(α)=1/2α are the most fitting mechanistic functions. The average E of 182.31 kJ/mol was close to the average value calculated by the FWO method (171.11 kJ/mol). The average A value of 5.37 × 10^10^ s^-1^ was greater than 1 × 10^9^ s^-1^, indicating the occurrence of simple complexation or interface reaction [41]. This finding aligns with previous studies on other fly ashes [24]. In addition, the E value is highly close to the activation energy of CaCO_3_ decomposition of 180.2 kJ/mol [42]. It is likely that Stage II may involve mainly combustion of carbon, carbonation of CaClOH and decomposition of minerals such as CaCO_3_. A different scenario was observed of Stage II in N_2_. The contracting sphere model, g(α)=1-(1-α)^1/3^, best characterized the decomposition process. This could be caused by the decomposition of CaClOH and CaCO_3_. This is the same as in the study by Halikia et al. [43]. In addition, the average A was less than 1 × 10^9^ s^-1^ indicating simpler reactions in this stage. This is consistence with results from kinetic analysis via model-free methods.

The three-dimensional diffusion model was identified as the optimal model for Stage III, with average E values of 386.92 kJ/mol (in air) and 372.08 kJ/mol (in N_2_), which are close with those obtained from the FWO method. Notably, these E values are significantly higher than the value of 277.99 kJ/mol reported by Zhao et al. [39] for fly ash in the same stage, while showing better consistency with the range of 363 kJ/mol-367 kJ/mol documented by Wang et al. [33]. The variations in E values can be primarily attributed to the inherent complexity and compositional heterogeneity of fly ash. Moreover, TG and kinetic analyses revealed that the mass loss and activation energy requirements were notably increased during Stage III. This phenomenon was due to the decomposition and transformation of CaSO_4_, CaCO_3_, NaCl, and KCl, etc. into complex crystalline structures. That is, the chemical bonds of these inorganic salts were destroyed in Stage III, causing the migration of formed free atoms or ions via surface or volume diffusion to uphold system stability [33]. This is consistent with the three-dimensional diffusion process. Additionally, the average A values during Stage III were above 1 × 10^14^ s^-1^ in both air and N_2_. The high value of A indicates prolonged reaction times and accelerated molecular collisions [44]. This further explained the heightened mass loss and reaction energy during this stage.

Based on the CR method’s calculations, the thermal decomposition kinetics equations of fly ash in air and N_2_ can be represented by Eq. (17)-(20).

Stage II in air:


$$\frac{d\alpha }{dT}=\frac{5.37\times 10^{10}}{\beta }\exp(-\frac{182.31}{RT})\times \frac{1}{(2}\alpha )$$ (17)

Stage III in air:


$$\frac{d\alpha }{dT}=\frac{1.57\times 10^{17}}{\beta }\exp(-\frac{386.92}{RT})\times \frac{3}{2}{\left(1-\alpha \right)}^{2/3}{\left[1-{\left(1-\alpha \right)}^{1/3}\right]}^{-1}$$ (18)

Stage II in N_2_:


$$\frac{d\alpha }{dT}=\frac{2.21\times 10^{7}}{\beta }\exp(-\frac{127.12}{RT})\times {3(1-\alpha )}^{2/3}$$ (19)

Stage III in N_2_:


$$\frac{d\alpha }{dT}=\frac{2.00\times 10^{16}}{\beta }\exp(-\frac{372.08}{RT})\times \frac{3}{2}{\left(1-\alpha \right)}^{2/3}{\left[1-{\left(1-\alpha \right)}^{1/3}\right]}^{-1}$$ (20)

**Fig 4:**
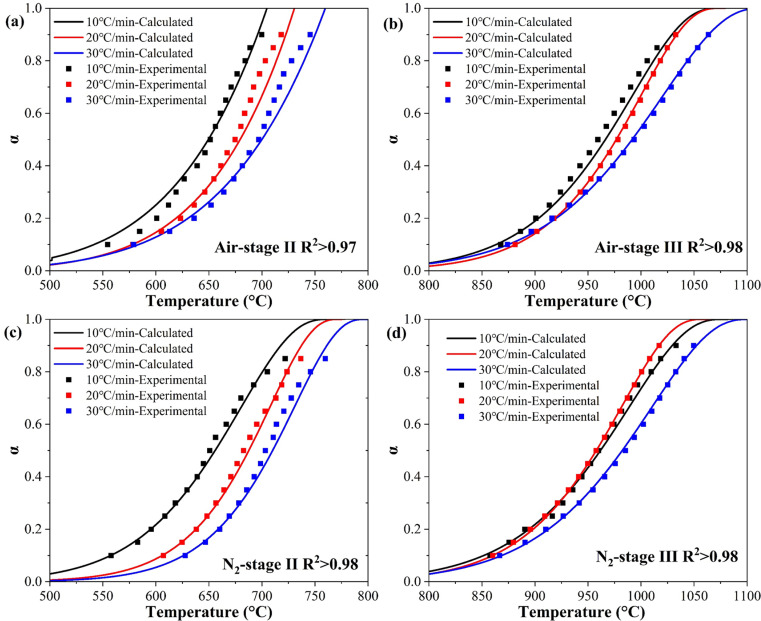
Compared the experimentalα with calculated α via each model.

Fig 4 compared the temperature-dependent relationships between calculated-α and experimental-α for each stage at heating rates of 10ºC/min, 20ºC/min, and 30ºC/min. Clearly, the fitting accuracy (R^2^ > 0.97) was consistently high for both calculated-α and experimental-α within each model. The results demonstrated the applicability of the one-dimensional diffusion and contracting sphere models in expressing the thermal reaction mechanisms of Stage II in air and N_2_, while the three-dimensional diffusion model was suitable for both Stage III.

### 3.5 Thermodynamic parameters analysis

**Fig 5:**
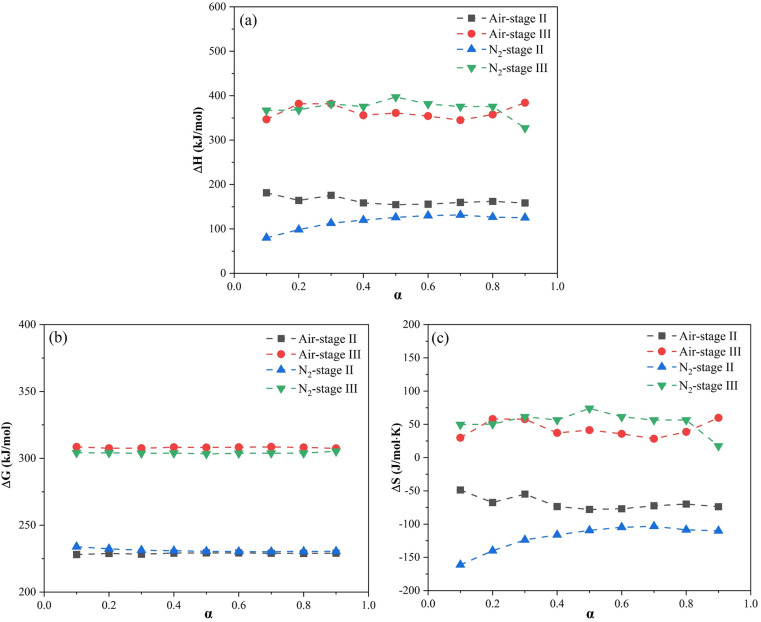
The relationship between conversion rate and the average values of (a) ΔH, (b) ΔG, and (c) ΔS of fly ash.

Thermodynamic parameters such as ΔH, ΔG, and ΔS of fly ash were estimated at heating rates of 10ºC/min, 20ºC/min, and 30ºC/min and documented in S5-S7 Tables. The trends in the changes of ΔH, ΔG, and ΔS within the range of 0.1 ≤ α ≤ 0.9 remained unaffected by alterations in heating rate. Consequently, their average values could provide a viable approach for investigating the thermal reaction of fly ash in air and N_2_. The variations of average ΔH, ΔG, and ΔS with α in the range of 0.1 to 0.9 were illustrated in Fig 5.

The parameter ΔH signifies the enthalpy difference between the products and reactants in a chemical reaction, with a positive value denoting an endothermic reaction [45]. For Stage II, the computed average ΔH values were 154.62 kJ/mol-181.43 kJ/mol in air and 79.88 kJ/mol-131.41 kJ/mol in N_2_. Comparatively, Stage III demonstrated higher values, ranging from 344.89 kJ/mol to 384.05 kJ/mol in air and from 327.37 kJ/mol to 396.98 kJ/mol in N_2_. These positive ΔH values were in accordance with the endothermic peaks observed in the DSC curves, indicating endothermic processes of both stages. The energy difference between the E and ΔH value (E-ΔH) is presented in S3 Fig. The average values of E-ΔH for Stage II were 7.73 kJ/mol and 7.81 kJ/mol in air and N_2_. Correspondingly, E-ΔH in Stage III were 10.27 kJ/mol in air and 10.20 kJ/mol in N_2_. The high energy barrier (>7 kJ/mol) observed in both stages may be attributable to the volatilization of elements and the decomposition of minerals, which induced changes in the system’s volume [39]. Moreover, Stage III necessitated a greater energy input than Stage II to overcome the activation barrier and achieve the desired chemical transformation.

The parameter ΔG, depicted in Fig 5 (b), serves as a thermodynamic indicator for assessing reaction directionality and feasibility. The ΔG values greater than zero signify non-spontaneous reactions [46]. Notably, ΔG values were stable across α values regardless of atmosphere suggesting an invariant reaction possibility throughout. The calculated ΔG ranged from 228.11 kJ/mol to 308.59 kJ/mol in air and 230.15 kJ/mol to 305.31 kJ/mol in N_2_, confirming that all reactions were not spontaneous and required extra energy for reactions to happen. In addition, Fig 5 (c) elucidated the fluctuation in ΔS, with its value serving as a metric for assessing system degree of disorder and reaction activity [47]. The negative ΔS values for Stage II implied a reduced disorder among the products, hinting at the enhanced stability of the fly ash structure post-thermal treatment [48]. Conversely, Stage III manifested elevated ΔG and positive ΔS values relative to Stage II in both atmospheres. These are indicative of the more challenging reaction and the high reaction activity at elevated temperatures [49]. The positive ΔS values also suggested an increase in the degree of system disorder, which is attributed to the enhanced release of elements during the later stage [50].

## 4. Conclusions

The chemical composition of fly ash was complex, consisting mainly of CaCO_3_, CaSO_4_, CaClOH, Ca(OH)_2_, KCl, NaCl, and SiO_2_. Thermogravimetric analysis revealed the total mass loss of fly ash ranged from 35.22% to 36.28% in air and from 36.83% to 46.38% in N_2_ within the temperature range of 30ºC to 1100ºC. The thermal decomposition process can be divided into three stages based on the mass loss: Stage I (30ºC-500ºC), Stage II (500ºC-800ºC), and Stage III (800ºC-1100ºC), with over 80% of the mass loss concentrated in the latter two stages.

Compared with KAS and Friedman methods, FWO method showed the highest correlation co-efficiency value explaining the kinetic data. Activation energies calculated based on FWO method were 171.11 kJ/mol for air and 124.52 kJ/mol in N_2_ for Stage II. Activation energies for reactions in Stage III were much higher than those in Stage II but were similar in values regardless of atmosphere (373.38 kJ/mol in air and 382.25 kJ/mol in N_2_). CR model further revealed that Stage II was controlled by one-dimensional diffusion (D1) in air and contracting sphere model (R3) in N_2_. Three-dimensional diffusion (D3) predominated in Stage III in both air and N_2_ probably because reactions such as volatilization of chloride salts and formation of complex compounds such as Ca_2_SiO_4_, (CaO)_12_(Al_2_O_3_)_7_, and Ca_10_(SiO_4_)_3_(SO_4_)_3_Cl_2_.

Thermodynamic analysis further revealed that the thermal reactions of fly ash were non-spontaneous and endothermic, with Stage III demonstrating higher complexity and reactivity compared to Stage II. These findings provided a rational explanation for the thermal behavior of fly ash in air and N_2_ and could provide robust benchmarks to design and optimize thermal treatment processes.

## Supporting information

S1 Fig Kinetic plots of Stages II-III in air atmosphere by FWO, KAS, and Friedman (FR) methods. (TIF)

S2 Fig Kinetic plots of Stages II-III in N_2_ atmosphere by FWO, KAS, and Friedman (FR) methods. (TIF)

S1-S4 Tables Values of E and R^2^ via CR method based on 15 reaction models. (DOCX)

S5-S7 Tables Thermodynamic parameters of fly ash thermal treated in air and N_2_ atmospheres. (DOCX)

S3 Fig The relationship between conversion rate and the difference between the E and ΔH (E-ΔH). (TIF)
