# Supplementary figures and images for: Study on kinetics and thermsodynamics of municipal solid waste incineration fly ash in air and N2 atmospheres
Source: PLoS One. 2025 May 14;20(5):e0323729. doi: 10.1371/journal.pone.0323729 (PMC12077739; doi:10.1371/journal.pone.0323729)

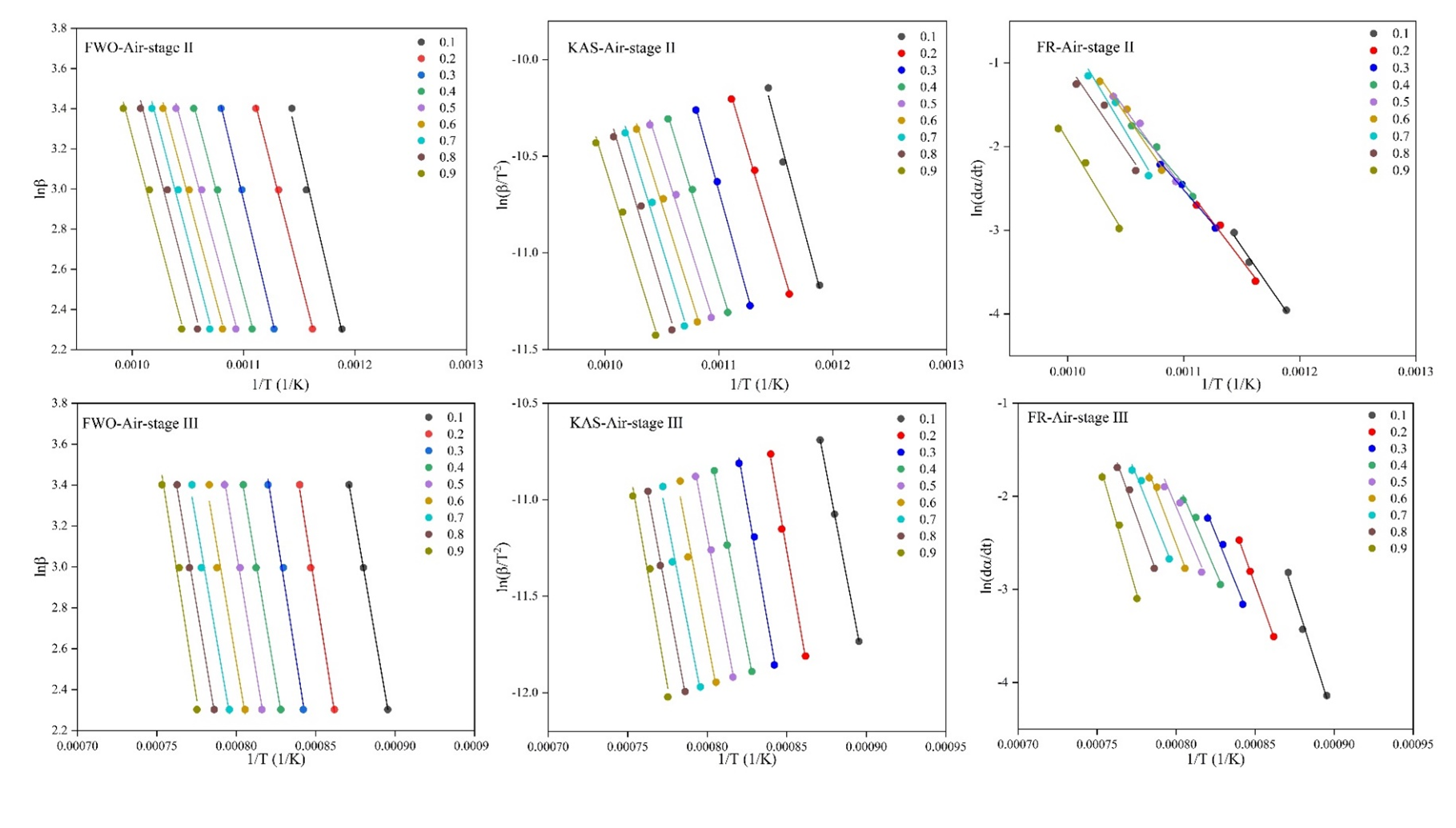

Supplement: S1 Fig — (TIF) [file pone.0323729.s001.tif]

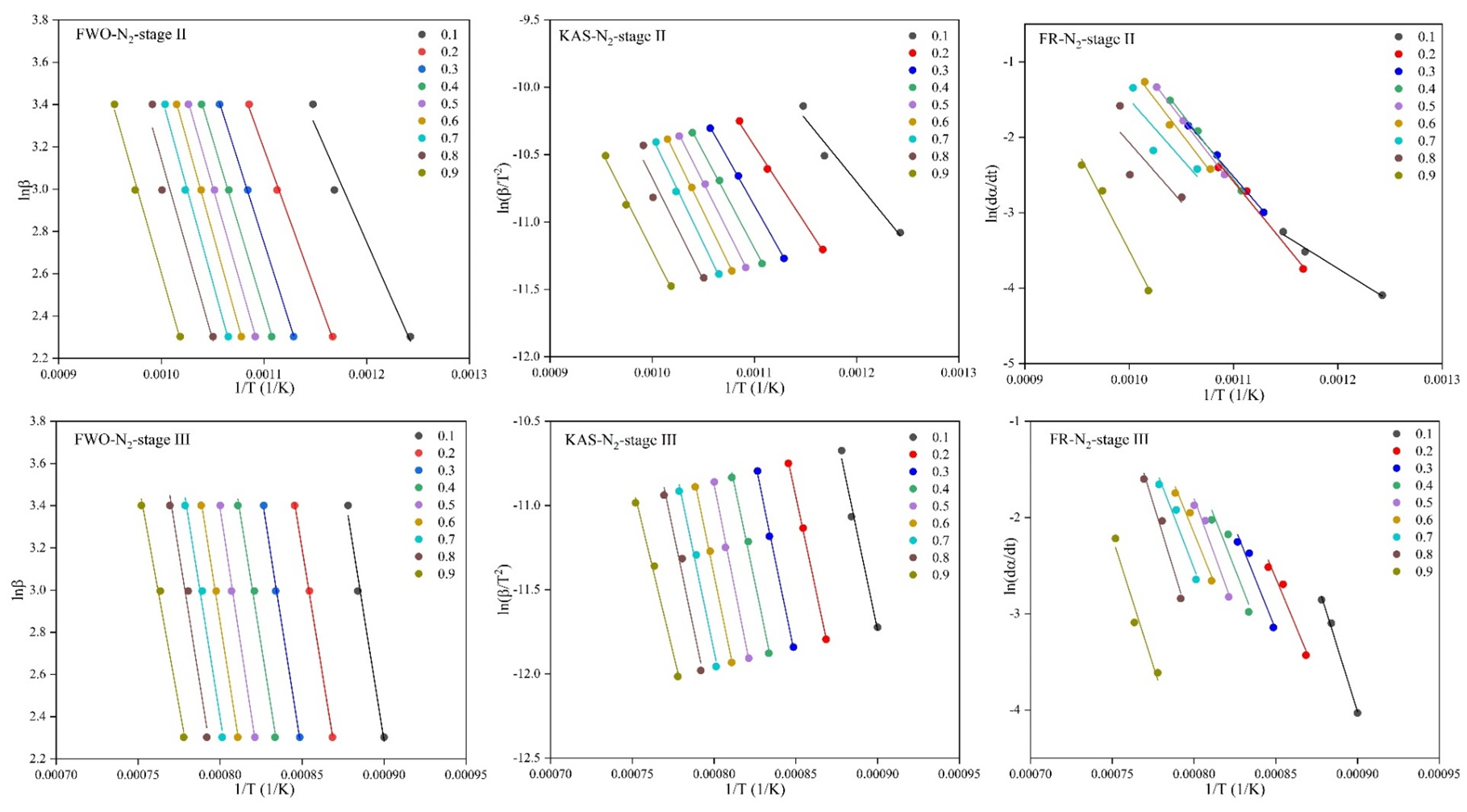

Supplement: S2 Fig — (TIF) [file pone.0323729.s002.tif]

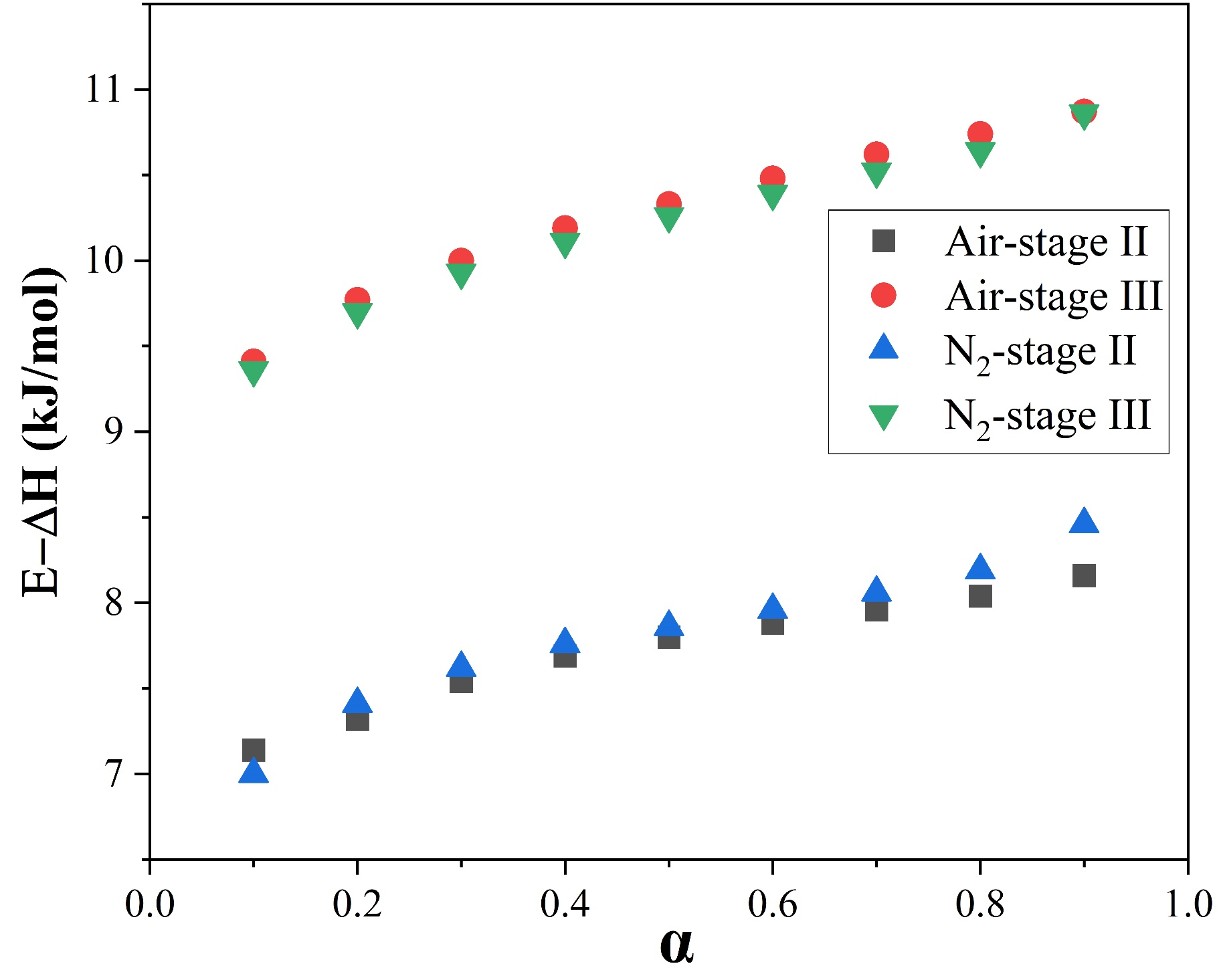

Supplement: S3 Fig — (TIF) [file pone.0323729.s005.tif]
