# Supplementary material for: Study on kinetics and thermsodynamics of municipal solid waste incineration fly ash in air and N2 atmospheres
Source: PLoS One. 2025 May 14;20(5):e0323729. doi: 10.1371/journal.pone.0323729 (PMC12077739; doi:10.1371/journal.pone.0323729)
Supplement: S1-S4 Tables — (DOCX) [file pone.0323729.s003.docx]

**S1 Table. Values of E (kJ/mol) and R^2^ in Stage II in air atmosphere via CR method based on 15 reaction models**

| **Code** | **10ºC/min** | | **20ºC/min** | | **30ºC/min** | | **Average** | |
| --- | --- | --- | --- | --- | --- | --- | --- | --- |
|  | **E** | **R^2^** | **E** | **R^2^** | **E** | **R^2^** | **E** | **R^2^** |
| F2 | 181.48 | 0.971 | 164.85 | 0.932 | 159.98 | 0.895 | 168.77 | 0.933 |
| F3 | 260.80 | 0.957 | 273.13 | 0.884 | 218.86 | 0.834 | 250.93 | 0.892 |
| R1 | 78.21 | 0.908 | 91.83 | 0.981 | 80.57 | 0.987 | 83.54 | 0.959 |
| R2 | 95.97 | 0.940 | 109.78 | 0.985 | 94.90 | 0.978 | 100.22 | 0.968 |
| R3 | 103.03 | 0.949 | 116.84 | 0.983 | 100.45 | 0.973 | 106.77 | 0.968 |
| D1 | 171.64 | 0.974 | 198.88 | 0.988 | 176.41 | 0.989 | 182.31 | 0.984 |
| D2 | 192.62 | 0.940 | 220.18 | 0.986 | 193.62 | 0.985 | 202.14 | 0.970 |
| D3 | 221.28 | 0.956 | 248.89 | 0.985 | 216.17 | 0.976 | 228.78 | 0.972 |
| D4 | 201.95 | 0.946 | 229.54 | 0.987 | 200.99 | 0.982 | 210.83 | 0.972 |
| A2 | 51.87 | 0.950 | 58.78 | 0.971 | 48.77 | 0.947 | 53.14 | 0.956 |
| A3 | 29.50 | 0.930 | 34.11 | 0.963 | 27.42 | 0.929 | 30.34 | 0.941 |
| A4 | 18.32 | 0.899 | 21.78 | 0.951 | 16.75 | 0.900 | 18.95 | 0.917 |
| P2 | 31.49 | 0.861 | 28.31 | 0.972 | 32.65 | 0.981 | 30.82 | 0.938 |
| P3 | 15.92 | 0.775 | 20.47 | 0.956 | 16.67 | 0.969 | 17.69 | 0.900 |
| P4 | 8.13 | 0.606 | 11.55 | 0.922 | 8.69 | 0.939 | 9.46 | 0.822 |

**S2 Table. Values of E (kJ/mol) and R^2^ in Stage III in air atmosphere via CR method based on 15 reaction models**

| **Code** | **10ºC/min** | | **20ºC/min** | | **30ºC/min** | | **Average** | |
| --- | --- | --- | --- | --- | --- | --- | --- | --- |
|  | **E** | **R^2^** | **E** | **R^2^** | **E** | **R^2^** | **E** | **R^2^** |
| F2 | 306.81 | 0.991 | 312.23 | 0.957 | 253.32 | 0.958 | 290.79 | 0.969 |
| F3 | 430.33 | 0.962 | 415.55 | 0.903 | 339.48 | 0.904 | 395.12 | 0.923 |
| R1 | 142.77 | 0.939 | 168.92 | 0.988 | 133.66 | 0.982 | 148.45 | 0.970 |
| R2 | 171.85 | 0.973 | 195.83 | 0.998 | 156.17 | 0.996 | 174.62 | 0.989 |
| R3 | 183.22 | 0.982 | 206.05 | 0.998 | 164.73 | 0.997 | 184.67 | 0.992 |
| D1 | 305.84 | 0.947 | 358.08 | 0.990 | 253.32 | 0.958 | 305.75 | 0.965 |
| D2 | 340.52 | 0.966 | 390.79 | 0.996 | 315.13 | 0.993 | 348.81 | 0.985 |
| D3 | 386.74 | 0.984 | 432.34 | 0.999 | 349.88 | 0.998 | 389.65 | 0.994 |
| D4 | 355.62 | 0.973 | 404.44 | 0.998 | 326.54 | 0.995 | 362.20 | 0.989 |
| A2 | 94.19 | 0.992 | 104.16 | 0.996 | 81.55 | 0.995 | 93.30 | 0.994 |
| A3 | 56.03 | 0.989 | 62.69 | 0.995 | 253.32 | 0.958 | 124.01 | 0.981 |
| A4 | 36.94 | 0.985 | 41.96 | 0.994 | 30.57 | 0.991 | 36.49 | 0.990 |
| P2 | 61.23 | 0.917 | 74.34 | 0.984 | 56.62 | 0.974 | 64.06 | 0.958 |
| P3 | 34.05 | 0.883 | 42.81 | 0.978 | 30.94 | 0.959 | 35.93 | 0.940 |
| P4 | 20.46 | 0.825 | 27.05 | 0.968 | 18.10 | 0.931 | 21.87 | 0.908 |

**S3 Table. Values of E (kJ/mol) and R2 in Stage II in N2 atmosphere via CR method based on 15 reaction models**

| **Code** | **10ºC/min** | | **20ºC/min** | | **30ºC/min** | | **Average** | |
| --- | --- | --- | --- | --- | --- | --- | --- | --- |
|  | **E** | **R^2^** | **E** | **R^2^** | **E** | **R^2^** | **E** | **R^2^** |
| F2 | 161.97 | 0.987 | 193.83 | 0.996 | 221.94 | 0.978 | 192.58 | 0.987 |
| F3 | 229.44 | 0.972 | 236.69 | 0.986 | 301.73 | 0.942 | 255.95 | 0.967 |
| R1 | 72.46 | 0.917 | 118.27 | 0.978 | 113.27 | 0.973 | 101.33 | 0.956 |
| R2 | 88.33 | 0.952 | 133.20 | 0.989 | 133.23 | 0.990 | 118.25 | 0.977 |
| R3 | 101.87 | 0.977 | 138.60 | 0.992 | 140.89 | 0.993 | 127.12 | 0.988 |
| D1 | 160.16 | 0.933 | 251.97 | 0.981 | 242.49 | 0.977 | 218.21 | 0.964 |
| D2 | 179.07 | 0.949 | 270.72 | 0.987 | 266.55 | 0.986 | 238.78 | 0.974 |
| D3 | 204.30 | 0.967 | 292.64 | 0.993 | 297.71 | 0.994 | 264.88 | 0.985 |
| D4 | 187.32 | 0.956 | 277.99 | 0.989 | 276.76 | 0.990 | 247.36 | 0.978 |
| A2 | 46.60 | 0.966 | 67.32 | 0.994 | 90.97 | 0.996 | 68.30 | 0.985 |
| A3 | 25.99 | 0.950 | 39.74 | 0.992 | 42.00 | 0.995 | 35.91 | 0.979 |
| A4 | 15.68 | 0.923 | 25.95 | 0.989 | 27.52 | 0.993 | 23.05 | 0.968 |
| P2 | 28.62 | 0.869 | 51.42 | 0.970 | 48.67 | 0.963 | 42.90 | 0.934 |
| P3 | 14.00 | 0.773 | 29.14 | 0.958 | 27.14 | 0.946 | 23.43 | 0.892 |
| P4 | 6.69 | 0.571 | 18.00 | 0.938 | 16.37 | 0.916 | 13.69 | 0.808 |

**S4 Table. Values of E (kJ/mol) and R^2^ in Stage III in N_2_ atmosphere via CR method based on 15 reaction models**

| **Code** | **10ºC/min** | | **20ºC/min** | | **30ºC/min** | | **Average** | |
| --- | --- | --- | --- | --- | --- | --- | --- | --- |
|  | **E** | **R^2^** | **E** | **R^2^** | **E** | **R^2^** | **E** | **R^2^** |
| F2 | 286.60 | 0.996 | 304.84 | 0.965 | 255.17 | 0.952 | 282.20 | 0.971 |
| F3 | 405.59 | 0.980 | 415.43 | 0.914 | 340.26 | 0.895 | 387.09 | 0.930 |
| R1 | 128.96 | 0.911 | 154.50 | 0.977 | 136.62 | 0.987 | 140.03 | 0.958 |
| R2 | 156.86 | 0.953 | 181.98 | 0.994 | 159.00 | 0.997 | 165.95 | 0.981 |
| R3 | 167.77 | 0.964 | 192.57 | 0.997 | 167.50 | 0.997 | 175.95 | 0.986 |
| D1 | 278.23 | 0.923 | 329.02 | 0.980 | 293.48 | 0.989 | 300.24 | 0.964 |
| D2 | 311.45 | 0.946 | 362.15 | 0.990 | 320.76 | 0.996 | 331.45 | 0.977 |
| D3 | 355.85 | 0.979 | 405.15 | 0.998 | 355.23 | 0.998 | 372.08 | 0.992 |
| D4 | 325.96 | 0.954 | 376.24 | 0.994 | 332.08 | 0.997 | 344.76 | 0.982 |
| A2 | 85.98 | 0.977 | 98.02 | 0.997 | 82.94 | 0.993 | 88.98 | 0.989 |
| A3 | 50.55 | 0.969 | 58.67 | 0.997 | 48.55 | 0.991 | 52.59 | 0.986 |
| A4 | 32.83 | 0.958 | 39.00 | 0.996 | 31.35 | 0.986 | 34.39 | 0.980 |
| P2 | 54.32 | 0.877 | 67.24 | 0.969 | 58.19 | 0.981 | 59.92 | 0.942 |
| P3 | 29.45 | 0.821 | 38.16 | 0.956 | 32.05 | 0.971 | 33.22 | 0.916 |
| P4 | 17.01 | 0.726 | 23.62 | 0.934 | 18.98 | 0.952 | 19.87 | 0.871 |
