# Supplementary material for: Study on kinetics and thermsodynamics of municipal solid waste incineration fly ash in air and N2 atmospheres
Source: PLoS One. 2025 May 14;20(5):e0323729. doi: 10.1371/journal.pone.0323729 (PMC12077739; doi:10.1371/journal.pone.0323729)
Supplement: S5-S7 Tables — (DOCX) [file pone.0323729.s004.docx]

**S5 Table. The ΔH (kJ/mol) of fly ash thermal treated in air and N_2_ atmospheres**

| **Stage** | **α** | **ΔH** | | | |
| --- | --- | --- | --- | --- | --- |
|  |  | **10ºC/min** | **20ºC/min** | **30ºC/min** | **Average** |
| II  (Air) | 0.1 | 181.58 | 181.39 | 181.31 | 181.43 |
|  | 0.2 | 164.41 | 164.22 | 164.08 | 164.23 |
|  | 0.3 | 175.93 | 175.74 | 175.61 | 175.76 |
|  | 0.4 | 158.80 | 158.59 | 158.43 | 158.61 |
|  | 0.5 | 154.83 | 154.61 | 154.44 | 154.62 |
|  | 0.6 | 155.82 | 155.60 | 155.42 | 155.61 |
|  | 0.7 | 159.91 | 159.70 | 159.51 | 159.71 |
|  | 0.8 | 162.21 | 162.00 | 161.81 | 162.01 |
|  | 0.9 | 158.66 | 158.43 | 158.23 | 158.44 |
| III  (Air) | 0.1 | 346.85 | 346.69 | 346.59 | 346.71 |
|  | 0.2 | 381.94 | 381.77 | 381.69 | 381.80 |
|  | 0.3 | 381.82 | 381.67 | 381.56 | 381.68 |
|  | 0.4 | 356.06 | 355.87 | 355.77 | 355.90 |
|  | 0.5 | 361.24 | 361.06 | 360.94 | 361.08 |
|  | 0.6 | 354.31 | 354.08 | 354.02 | 354.14 |
|  | 0.7 | 345.07 | 344.84 | 344.76 | 344.89 |
|  | 0.8 | 357.88 | 357.67 | 357.56 | 357.71 |
|  | 0.9 | 384.20 | 384.05 | 383.89 | 384.05 |
| II  (N_2_) | 0.1 | 80.21 | 79.78 | 79.65 | 79.88 |
|  | 0.2 | 98.76 | 98.41 | 98.22 | 98.46 |
|  | 0.3 | 113.26 | 112.95 | 112.75 | 112.99 |
|  | 0.4 | 120.11 | 119.81 | 119.61 | 119.84 |
|  | 0.5 | 126.16 | 125.87 | 125.68 | 125.90 |
|  | 0.6 | 130.33 | 130.03 | 129.85 | 130.07 |
|  | 0.7 | 131.67 | 131.35 | 131.19 | 131.41 |
|  | 0.8 | 126.88 | 126.48 | 126.40 | 126.59 |
|  | 0.9 | 125.57 | 125.20 | 125.02 | 125.26 |
| III  (N_2_) | 0.1 | 367.02 | 366.85 | 366.78 | 366.88 |
|  | 0.2 | 368.06 | 367.90 | 367.79 | 367.92 |
|  | 0.3 | 381.67 | 381.50 | 381.41 | 381.52 |
|  | 0.4 | 375.59 | 375.43 | 375.31 | 375.44 |
|  | 0.5 | 397.13 | 396.95 | 396.87 | 396.98 |
|  | 0.6 | 381.55 | 381.38 | 381.26 | 381.40 |
|  | 0.7 | 375.64 | 375.48 | 375.34 | 375.48 |
|  | 0.8 | 375.65 | 375.50 | 375.34 | 375.50 |
|  | 0.9 | 327.56 | 327.36 | 327.19 | 327.37 |

**S6 Table. The ΔG (kJ/mol) of fly ash thermal treated in air and N_2_ atmospheres**

| **Stage** | **α** | **ΔG** | | | |
| --- | --- | --- | --- | --- | --- |
|  |  | **10ºC/min** | **20ºC/min** | **30ºC/min** | **Average** |
| II  (Air) | 0.1 | 221.26 | 228.89 | 234.17 | 228.11 |
|  | 0.2 | 221.99 | 229.65 | 234.94 | 228.86 |
|  | 0.3 | 221.48 | 229.12 | 234.40 | 228.33 |
|  | 0.4 | 222.23 | 229.89 | 235.19 | 229.11 |
|  | 0.5 | 222.41 | 230.08 | 235.38 | 229.29 |
|  | 0.6 | 222.36 | 230.03 | 235.33 | 229.24 |
|  | 0.7 | 222.17 | 229.83 | 235.13 | 229.04 |
|  | 0.8 | 222.06 | 229.72 | 235.01 | 228.93 |
|  | 0.9 | 222.22 | 229.88 | 235.18 | 229.09 |
| III  (Air) | 0.1 | 303.17 | 308.21 | 314.32 | 308.57 |
|  | 0.2 | 302.18 | 307.20 | 313.30 | 307.56 |
|  | 0.3 | 302.17 | 307.20 | 313.29 | 307.55 |
|  | 0.4 | 302.88 | 307.92 | 314.02 | 308.27 |
|  | 0.5 | 302.73 | 307.76 | 313.87 | 308.12 |
|  | 0.6 | 302.92 | 307.96 | 314.07 | 308.32 |
|  | 0.7 | 303.19 | 308.23 | 314.34 | 308.59 |
|  | 0.8 | 302.81 | 307.85 | 313.95 | 308.21 |
|  | 0.9 | 302.09 | 307.11 | 313.20 | 307.47 |
| II  (N_2_) | 0.1 | 226.98 | 233.18 | 241.56 | 233.91 |
|  | 0.2 | 225.45 | 231.62 | 239.94 | 232.34 |
|  | 0.3 | 224.45 | 230.58 | 238.88 | 231.30 |
|  | 0.4 | 224.01 | 230.14 | 238.42 | 230.86 |
|  | 0.5 | 223.65 | 229.77 | 238.03 | 230.48 |
|  | 0.6 | 223.41 | 229.52 | 237.78 | 230.23 |
|  | 0.7 | 223.33 | 229.44 | 237.69 | 230.15 |
|  | 0.8 | 223.59 | 229.71 | 237.97 | 230.42 |
|  | 0.9 | 223.65 | 229.77 | 238.03 | 230.48 |
| III  (N_2_) | 0.1 | 298.36 | 302.86 | 311.34 | 304.19 |
|  | 0.2 | 298.32 | 302.82 | 311.30 | 304.15 |
|  | 0.3 | 297.95 | 302.44 | 310.91 | 303.77 |
|  | 0.4 | 298.11 | 302.60 | 311.08 | 303.93 |
|  | 0.5 | 297.54 | 302.03 | 310.49 | 303.35 |
|  | 0.6 | 297.94 | 302.43 | 310.90 | 303.76 |
|  | 0.7 | 298.10 | 302.59 | 311.06 | 303.92 |
|  | 0.8 | 298.09 | 302.59 | 311.06 | 303.91 |
|  | 0.9 | 299.46 | 303.98 | 312.49 | 305.31 |

**S7 Table. The ΔS (J/(mol·K)) of fly ash thermal treated in air and N_2_ atmospheres**

| **Stage** | **α** | **ΔS** | | | |
| --- | --- | --- | --- | --- | --- |
|  |  | **10ºC/min** | **20ºC/min** | **30ºC/min** | **Average** |
| II  (Air) | 0.1 | -42.66 | -49.53 | -53.99 | -48.73 |
|  | 0.2 | -61.91 | -68.22 | -72.37 | -67.50 |
|  | 0.3 | -48.97 | -55.65 | -60.05 | -54.89 |
|  | 0.4 | -68.19 | -74.35 | -78.40 | -73.65 |
|  | 0.5 | -72.66 | -78.69 | -82.67 | -78.01 |
|  | 0.6 | -71.54 | -77.60 | -81.61 | -76.92 |
|  | 0.7 | -66.93 | -73.12 | -77.22 | -72.42 |
|  | 0.8 | -64.35 | -70.60 | -74.76 | -69.90 |
|  | 0.9 | -68.34 | -74.49 | -78.58 | -73.80 |
| III  (Air) | 0.1 | 34.61 | 30.04 | 24.74 | 29.80 |
|  | 0.2 | 63.20 | 58.20 | 52.44 | 57.95 |
|  | 0.3 | 63.11 | 58.13 | 52.34 | 57.86 |
|  | 0.4 | 42.14 | 37.43 | 32.01 | 37.19 |
|  | 0.5 | 46.36 | 41.60 | 36.09 | 41.35 |
|  | 0.6 | 40.72 | 36.00 | 30.63 | 35.78 |
|  | 0.7 | 33.19 | 28.57 | 23.32 | 28.36 |
|  | 0.8 | 43.63 | 38.89 | 33.44 | 38.65 |
|  | 0.9 | 65.06 | 60.05 | 54.20 | 59.77 |
| II  (N_2_) | 0.1 | -157.97 | -161.11 | -164.68 | -161.25 |
|  | 0.2 | -136.36 | -139.90 | -144.15 | -140.14 |
|  | 0.3 | -119.67 | -123.54 | -128.29 | -123.83 |
|  | 0.4 | -111.83 | -115.87 | -120.84 | -116.18 |
|  | 0.5 | -104.92 | -109.11 | -114.28 | -109.44 |
|  | 0.6 | -100.18 | -104.48 | -109.78 | -104.81 |
|  | 0.7 | -98.64 | -103.01 | -108.32 | -103.33 |
|  | 0.8 | -104.09 | -108.41 | -113.48 | -108.66 |
|  | 0.9 | -105.56 | -109.82 | -114.95 | -110.11 |
| III  (N_2_) | 0.1 | 55.10 | 50.66 | 42.81 | 49.52 |
|  | 0.2 | 55.96 | 51.52 | 43.62 | 50.37 |
|  | 0.3 | 67.18 | 62.58 | 54.43 | 61.40 |
|  | 0.4 | 62.17 | 57.66 | 49.60 | 56.48 |
|  | 0.5 | 79.92 | 75.15 | 66.69 | 73.92 |
|  | 0.6 | 67.09 | 62.50 | 54.32 | 61.31 |
|  | 0.7 | 62.22 | 57.70 | 49.63 | 56.52 |
|  | 0.8 | 62.24 | 57.72 | 49.63 | 56.53 |
|  | 0.9 | 22.54 | 18.51 | 11.35 | 17.47 |
